# A case series discussing the anaesthetic management of pregnant patients with brain tumours

**DOI:** 10.12688/f1000research.2-92.v2

**Published:** 2013-12-11

**Authors:** Alaa A Abd-Elsayed, Jose Díaz-Gómez, Gene H Barnett, Andrea Kurz, Maria Inton-Santos, Sabri Barsoum, Rafi Avitsian, Zeyd Ebrahim, Vesna Jevtovic-Todorovic, Ehab Farag

**Affiliations:** 1 Department of Anesthesiology, University of Cincinnati, Cincinnati, OH, 45267, USA; 2 Department of Outcomes Research, Anesthesia Institute, Cleveland Clinic, Cleveland, OH, 44195, USA; 3 Department of General Anesthesia, Cleveland Clinic, Cleveland, OH, 44195, USA; 4 Neurological Surgery and Rose Ella Burkhardt Brain Tumor & Neuro-Oncology Center, Cleveland Clinic, Cleveland, OH, 44195, USA; 5 Obstetrics & Gynecology Anesthesia, Hillcrest Hospital, Cleveland, OH, 44195, USA; 6 Anesthesiology, University of Virginia Health System, Charlottesville, VA, 22093, USA

## Abstract

Pregnancy may aggravate the natural history of an intracranial tumour, and may even unmask a previously unknown diagnosis. Here we present a series of seven patients who had brain tumours during pregnancy. The aim of this case series is to characterize the current perioperative management and to suggest evidence based guidelines for the anaesthetic management of pregnant females with brain tumours. This is a retrospective study. Information on pregnant patients diagnosed with brain tumours that underwent caesarean section (CS) and/or brain tumour resection from May 2003 through June 2008 was obtained from the Department of General Anaesthesia and the Rose Ella Burkhardt Brain Tumour & Neuro-Oncology Centre (BBTC) at the Cleveland Clinic, OH, USA. The mean age was 34.5 years (range 29-40 years old). Six patients had glioma, two of whom had concomitant craniotomy and CS. Six cases had the tumour in the frontal lobe. Four cases were operated on under general anaesthesia and three underwent awake craniotomy. The neonatal outcomes of the six patients with elective or emergent delivery were six viable infants with normal Apgar scores. Pregnancy was terminated in the 7th patient. In conclusion, good knowledge of the variable anesthetic agents and their effects on the fetus is very important in managing those patients.

## Introduction

Pregnancy may increase the growth of a previously existing intracranial tumour, and can even unmask a previously undiscovered tumour. A previous study that included 8 patients who had been diagnosed antenatally with a malignant brain tumour stated that all had severe neurological manifestations, and six of them had a severe neurological event that lead to premature termination of the pregnancy ^1^. It was suggested that immunological tolerance and steroid mediated growth led to this exacerbation during pregnancy ^2^.

In a population based study; Haas *et al.* reported that the number of meningiomas, acoustic neuromas, and primary malignant intracranial neoplasms diagnosed during pregnancy was less than expected with the ratio of observed/expected tumours associated with pregnancy to be 0.38 ^3^.

In 1988, Simon ^4^ postulated a theory to predict the prevalence of brain tumours in pregnant patients by using the intersection of the probability of being pregnant at any given time with the probability of having a brain tumour at a specific age and sex. Based on this theory the author calculated that in the USA there are about 89 pregnant women per year that also have brain tumours.

Brain tumours in pregnant patients impose a unique risk to both the foetus and mother. There are no previous studies that proposed any guidelines for the anaesthetic management of pregnant patients with brain tumours.

The aim of this case series is to characterize the current perioperative management of pregnant patients with brain tumours and to suggest guidelines for the proper anaesthetic management.

## Methods

Information on pregnant patients diagnosed with brain tumours that underwent CS and/or brain tumour resection from May 2003 to June 2008 was obtained from the Department of General Anaesthesia and the Rose Ella Burkhardt Brain Tumour & Neuro-Oncology Centre (BBTC) IRB-approved databases at the Cleveland Clinic in OH, USA. Patients were managed by the Departments of Neurosurgery, Obstetrics and Gynaecology, Anaesthesiology and BBTC. We used the Anaesthesia Record Keeping System (ARKS) to obtain the electronic record of the anaesthetic management. Additional data from the patients’ electronic and paper charts were used to complete the pre- and post-operative patient information.

## Results

Five pregnant patients presented with brain tumours during their pregnancy. An additional two patients had their diagnosis of brain tumours made in the immediate postpartum period. Diagnoses ( Table 1) included meningioma (1 patient) and glioma (6 patients). The mean age was 34.5 years (range 29–40 years) and parity was 0 (2 patients), 1 (1 patient), and >2 (4 patients). More than half of the patients (57%) underwent CS with craniotomy performed, on average, 45 days after the CS (range: 2–90 days). Two patients were diagnosed with a brain tumour during pregnancy and had a craniotomy ( Table 2). All our patients were managed by general anaesthesia or monitored anaesthesia care (MAC). Inhalational anaesthetic agents (isoflurane and desflurane) were used under 1-minimal alveolar concentration for the maintenance of anaesthesia. Four drugs were used in our patients for both induction and maintenance of anaesthesia; propofol in 2 pregnant patients, remifentanil in 2 pregnant patients and alfentanil in one patient. Foetal heart rate monitoring was applied in one patient receiving MAC for an “awake” craniotomy. Rapid sequence induction was not universally applied. Four cases had general anaesthesia and used rocuronium as a muscle relaxant to facilitate endotracheal intubation. There were no major intraoperative events ( Table 1 and Table 3). The neonatal outcomes of the six patients with elective or emergent delivery were six viable infants with normal Apgar scores. Pregnancy was terminated in the 7 ^th^ patient. There was neither operative mortality nor significant sustained morbidity in this series. One patient suffered a pulmonary embolus in the postoperative period.

**Table 1.** Anaesthetic techniques used for brain tumour resection at the Cleveland Clinic, Ohio (2003–2008).

Case	Anaesthesia technique	Anaesthesia induction	Anaesthesia maintenance	Postoperative events	Pathology
1	General	Propofol/fentanyl succinylcholine	Isoflurane	Pulmonary Embolism	Tentorial meningioma
2	General	Propofol/fentanyl Rocuronium	Desflurane-remifentanil	Seizures	Recurrent anaplastic glioma
3	General	Thiopental/fentanyl Rocuronium	Isoflurane-remifentanil	Medical termination of pregnancy	Anaplastic glioma
4	Awake craniotomy	Propofol/alfentanil	Propofol/alfentanil	Deceased 16 months after craniotomy	Glioma
5	Awake craniotomy	Propofol/ dexmedetomidine	Propofol/ dexmedetomidine	Expressive aphasia	Low grade glioma
6	Awake craniotomy	Propofol/ dexmedetomidine	Propofol/ dexmedetomidine	Mild aphasia	Low grade glioma
7	General	Propofol/fentanyl succinylcholine	Isoflurane-remifentanil	Deceased 36 months after craniotomy	Glioblastoma multiforme

*FHR: Foetal Heart Rate

**Table 2.** Preoperative information of pregnant patients with brain tumours at the Cleveland Clinic, Ohio (2003–2008).

Case	Age	Gestational age at the time of diagnosis (weeks)	Preoperative anticonvulsant medications	CS followed by tumour excision	Time after delivery for craniotomy (days)	Gestational age at craniotomy (weeks)	Presentation	Size and tumour localization	Newborn condition
1	37	33	Lorazepam	Yes	2	33	Increased ICP*, headache	Right Temporal 6 × 4.5 cm	Normal Apgar scores
2	36	36	Lorazepam	No	Concomitant craniotomy and C-section	N/A	Increased ICP, headache	Left Frontal 5 × 4 cm	Normal Apgar scores
3	29	6	Phenytoin	No	Craniotomy during pregnancy	12	Seizures	Left Frontal 5.9 × 3.3 cm	-
4	40	18	-	No	Craniotomy during pregnancy	22	Seizures	Left Frontal 2.4 × 2.2 cm	Normal Apgar scores, Perioperative FHR* monitoring
5	30	18	-	Yes	30	N/A	Seizures	Left Frontal 7.5 × 4.5 cm	Normal Apgar scores
6	34	Postpartum- 1 week	-	Yes	60	N/A	Seizures	Right frontal 6.1 × 4.7 cm	Normal Apgar scores
7	36	Post-partum – 3 weeks	Phenytoin	Yes	90 (pregnancy was terminated)	N/A	Seizures and focal signs	Right frontal 2.8 × 2.3 cm	Normal Apgar scores

*ICP: Intra-Cranial Pressure

**Table 3.** Intraoperative management for brain tumour resection at the Cleveland Clinic, Ohio (2003–2008).

Case	Intraoperative hypotension	*EBL (ml)	Use of colloids (ml)	Total intravenous fluids (ml)	Use of furosemide	Urine output (ml)	Highest BP (mmHg)	**ETCO2 range (mmHg)
1	Yes	2200	1000	7500	Yes	2760	150/100	25–40
2	Yes	100	0	1800	No	790	140/80	22–43
3	Yes	1000	1000	4700	Yes	1700	150/80	26–38
4	Yes	500	0	5600	No	3700	120/70	26–34
5	No	100	0	4500	No	4400	170/90	N/A
6	No	400	0	4000	No	1045	160/100	20–30
7	No	100	0	3400	No	1800	140/60	22–36

*EBL: Estimated Blood Loss

** ETCO2: End Tidal CO2

## Discussion

Brain tumours tend to increase in size during pregnancy due to several factors such as fluid retention, increased blood volume and hormonal changes and therefore may be diagnosed earlier ^5^. The decision to proceed with neurosurgery during pregnancy depends on the site, size, type of tumour, neurological signs and symptoms, age of the foetus, and the patient’s wishes ^6, 7^.

**Figure 1.**
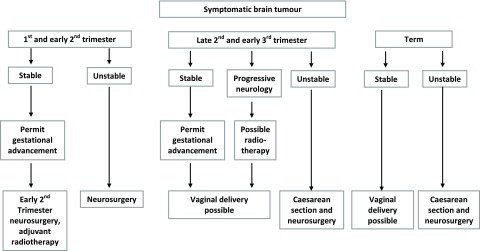
Algorithm for management of brain tumours in pregnant women.

There are no guidelines for the management of intracranial tumours in pregnant women. A possible algorithm to follow is shown in Figure 1 (modified from Tewari *et al.* ^1^).

### Management issues

Corticosteroids have been recommended as they are safe in pregnancy, promote foetal lung maturity and reduce cerebral oedemas ^1^.

During the first and early second trimesters, if the patient is stable, it is acceptable to permit pregnancy to proceed into the early second trimester and surgery can then be performed at this time. It is also possible to administer radiotherapy, radio-surgery and image guided surgery beyond the first trimester. If the patient is unstable, undergoing an urgent neurosurgery is recommended ^1^.

At the end of the second trimester; in stable patients, proceed with pregnancy with close observation. But if the patient has a worsening neurological status; radiotherapy can be used to delay surgery. If the patient is unstable and shows symptoms of impending herniation, it is recommended to use general anaesthesia to deliver the baby by CS which is followed by surgical decompression ^1^.

At term; in a stable patient, induction of vaginal delivery is permitted ^8^. A shortened second stage can be achieved with epidural anaesthesia ^9^. CS should only be performed for accepted indications as it has been shown that CS does not seem to provide any advantage over vaginal delivery in protecting against increased intracranial pressure. In unstable patients, perform, as above, CS under general anaesthesia, followed by surgical decompression ^1^.

Mannitol and hypocapnia were avoided in our patients to prevent foetal dehydration and cerebral ischemia/hypoxia, respectively.

General anaesthesia can be safe in pregnant patients with intracranial tumours. Tracheal intubation is very important as it allows maternal hyperventilation thereby controlling raised intracranial pressure ^10^. Patients should be pre-medicated with ranitidine 50 mg I.V. to protect the patient against possible vomiting and aspiration.

Propofol was used in 2 pregnant patients without producing any side effects. The main side effect is that it has a relaxing effect on the gravid uterus ^11^. It is still controversial whether its use is safe with newborns. Bacon *et al.* did not report any adverse effects of propofol in newborns after emergency CS ^12^ while another study reported seizure, ataxia, and hallucinations after prolonged propofol anaesthesia for more than 6 hours ^13^.

Meanwhile, isoflurane is known to produce many adverse effects on the foetus ^4, 5^. It was used in one of our pregnant patients but our records did not show any adverse effects. Desflurane was used in one of our patients with no complications. But the neurotoxicity of desflurane and sevoflurane is still a controversial issue ^16^.

Remifentanil was used in 2 pregnant patients without producing any adverse effects; this may be explained by the fact that it has a unique metabolism by plasma and tissue esterases and a context-sensitive half-life of 3 to 4 min, independent of the duration of infusion ^17^. One concern is that the transfer of opioids, such as remifentanil, across the placenta may lead to neonatal depression. However, remifentanil can be metabolized and redistributed to both the mother and the foetus rapidly ^18^. Remifentanil has opioid properties that allow both control of the intraoperative stress response and a more rapid recovery compared to other opioids. Because of its metabolism and short duration of action, remifentanil is therefore considered to be safe and effective for general anaesthesia for emergency CS in patients with neurological risk factors ^19^.

Clinically relevant concentrations of remifentanil induce rapid, persistent increases in NMDA-induced ion currents. Since NMDA-receptor blockade during a critical stage in brain development leads to depression of neuronal activity and as such is known to initiate the apoptotic cell death cascade in immature neurons, we suggest that remifentanil may be safe for the developing brain. In addition, remifentanil is known to offer a neuron-protective effect in cases of opioid induced hyperalgesia or tolerance ^20^. Dexmedetomidine use is recommended in pregnant patients ^21^.

### Possible medications for cases of brain tumours during pregnancy not used in this case series

In the following paragraphs we will discuss drugs that were not used in our study but have been investigated before.

There are no human trials examining the effects of nitrous oxide on neuronal structure and neurocognitive performance in young children. Some case studies showed that the exposure of neonates to nitrous oxide *in utero* during the third trimester or during CS can result in transient neurological sequelae ^22^.

Although sevoflurane is one of the most prevalent volatile anaesthetics, a recent study has suggested that it can cause epileptic seizure activity, neurotoxicity, and both acute and chronic impairment in synaptic plasticity in neonatal rats ^16^.

Oxytocin has been used in patients with intracranial tumours without any adverse effects. Ergotamine can cause hypertensive responses, which may increase the intracranial cranial pressure and can lead to haemorrhage. It should be avoided in pregnant women with brain tumours ^3^.

Dexamethasone has been traditionally used to reduce brain oedema. It may be safe to use it in an acute setting but its chronic use may be harmful to the foetus as it may cause hypoadrenalism. Weighting the risks and benefits for treating seizures with anticonvulsants; it is recommended to use them in this setting to avoid seizures that may lead to maternal and foetal hypoxia and acidosis ^23^.

Several studies investigated the mechanism of anaesthesia-induced neurotoxicity. Previous reports suggested depression of neuronal activity due to anaesthesia induced GABA A receptor activation and NMDA receptor blockade during a critical stage in brain development ^20^. Several adjuvants, such as estradiol, pilocarpine, melatonin and dexmedetomidine, have been identified in animal studies to ameliorate anaesthesia induced neurodegeneration ^24– 26^. It is still controversial whether etomidate is neurotoxic or not. There is evidence that the rarely used anaesthetic, xenon, in clinical doses does not have neurodegenerative effects and may be neuroprotective ^27^.

A recent study showed that the administration of lithium significantly increased the activation of a neuroprotective pathway in the hippocampus. Further studies and human trials are necessary to fully investigate the beneficial effects of lithium in the anaesthetic management of pregnant patients with brain tumours ^28^.

## Conclusion

Management of brain tumours in pregnant women is mainly reliant on case reports and the doctor’s personal experience. Therefore, close communication between the neurosurgeon, neuroanaesthetist, obstetrician and the patient is crucial. Good knowledge of the variable anesthetic agents and their effects on the fetus is very important in managing those patients.
