# Supplementary material for: A case series discussing the anaesthetic management of pregnant patients with brain tumours
Source: F1000Res. 2013 Dec 11;2:92. Originally published 2013 Mar 21. [Version 2] doi: 10.12688/f1000research.2-92.v2 (PMC3814915; doi:10.12688/f1000research.2-92.v2)
Supplement: Peer review history

# Peer review: Referee response for version 2

**Published:** 2014-01-08

**Authors:** Carolyn Weiniger

**Affiliations:** 1 Anesthesiology and Critical Care, Hadassah-University Medical Center, Jerusalem, Israel

This paper presents a small case series of pregnant women undergoing intracranial surgery, in addition to two postpartum neurosurgical patients. The paper is now much improved for clarity regarding patients who were actually pregnant during the surgery, and provides a short review of possible anesthetic management strategies for these patients.

I have read this submission. I believe that I have an appropriate level of expertise to confirm that it is of an acceptable scientific standard.

# Peer review: Referee response for version 2

**Published:** 2013-12-18

**Authors:** Michael James Paech

**Affiliations:** 1 School of Medicine and Pharmacology, University of Western Australia, Perth, Australia

I have read this submission. I believe that I have an appropriate level of expertise to confirm that it is of an acceptable scientific standard.

# Peer review: Referee response for version 1

**Published:** 2013-10-24

**Authors:** Carolyn Weiniger

**Affiliations:** 1 Anesthesiology and Critical Care, Hadassah-University Medical Center, Jerusalem, Israel

The case series reports brain tumors and cesarean delivery (CD) over a five year period in Cleveland Clinics, Ohio. The stated aim was to elucidate management suggestions from previously performed cases in their institution.

The case series includes seven patients; 5 with preCD and 2 with postCD diagnosis. The results state that "over half patients underwent CD with craniotomy up to 45 days post CD". Only one woman had craniotomy during pregnancy. Tables 1 and 2 somewhat clarify this confusing information. The authors report in the results that one patient had tumor diagnosed during pregnancy and had peripartum craniotomy, yet table 2 reports three such cases.

Table 1 reports the anesthesia drugs used for craniotomy surgery in the seven patients. Since only 3 patients had craniotomy with CD, it is not clear why Apgar scores are reported here for the patients who had post CD craniotomy.

Three patients, including one at 22 weeks gestation and two postCD had awake craniotomys. The authors report that fetal monitoring was used in only one of the awake craniotomy patients. It is my understanding that the other two had already delivered at time of craniotomy. The stated study aim was to provide management suggestions for anesthetic management of pregnant patients with brain tumors. I assumed this meant management for the CD. There are few details regarding the CD surgery, and four patients undergoing craniotomy were no longer pregnant at time of the craniotomy. Therefore table 3 also doesn't make sense- it is titled "management of pregnant patients for brain tumor resection" - again, four patients are no longer pregnant.

The authors present an algorithm for delivery of women with brain tumors. I could not find in their results any case of VD, all seemed to have CD. I wonder why the two postpartum diagnosed patients had CD? Their tumor was discovered 1 and 3 weeks postpartum.

The authors mention that VD is a delivery option, but their study does not bring new evidence for this. The algorithm combines information from other studies and is quite clear, but I wonder if their series adds to our knowledge. For example, stating that propofol was used in six patients and is controversial with newborns is not relevant for the four women who had craniotomy postpartum. Same goes for isoflurane and later - dexmetotomidine. The literature review of pharmacological agents is nicely presented. However again the conclusion reporting safety of des/iso is not particularly relevant, as some women were not pregnant when the drugs were given.

I have read this submission. I believe that I have an appropriate level of expertise to confirm that it is of an acceptable scientific standard, however I have significant reservations, as outlined above.

# Response

**Published:** 2013-12-11

**Authors:** Alaa Abd-Elsayed

**Affiliations:** Community Medicine, Faculty of Medicine, Assiut University, Egypt

We would like to thank the reviewer for her valid points.

- " *The authors report in the results that one patient had tumor diagnosed during pregnancy and had peripartum craniotomy, yet table 2 reports three such cases*"
  We have changed this to two patients, as the third case had both managed together.
   
- " *Table 1 reports the anesthesia drugs used for craniotomy surgery in the seven patients. Since only 3 patients had craniotomy with CD, it is not clear why Apgar scores are reported here for the patients who had post CD craniotomy.*"
  We wanted to show the outcomes of all pregnancies regardless of the final management, and we think it is important to mention the fetal outcome.
   
- " *There are few details regarding the CD surgery, and four patients undergoing craniotomy were no longer pregnant at time of the craniotomy. Therefore table 3 also doesn't make sense- it is titled "management of pregnant patients for brain tumor resection" - again, four patients are no longer pregnant*"
  We agree with the reviewer - we still think it is important to have this table, but maybe we can change the title to "Intraoperative management".
   
- *"The authors present an algorithm for delivery of women with brain tumors. I could not find in their results any case of VD, all seemed to have CD. I wonder why the two postpartum diagnosed patients had CD?"*
  That is a very good point. We wanted to include the algorithm as part of our discussion, as it serves very well to help people managing their cases with the same disease. Our article mainly carries the message of what we did and what can be done better.
   
- " *The authors mention that VD is a delivery option, but their study does not bring new evidence for this. The algorithm combines information from other studies and is quite clear, but I wonder if their series adds to our knowledge. For example, stating that propofol was used in six patients and is controversial with newborns is not relevant for the four women who had craniotomy postpartum. Same goes for isoflurane and later - dexmetotomidine. The literature review of pharmacological agents is nicely presented. However again the conclusion reporting safety of des/iso is not particularly relevant, as some women were not pregnant when the drugs were given.*"
  We agree with the reviewer, and have changed this in the manuscript.

# Peer review: Referee response for version 1

**Published:** 2013-10-07

**Authors:** Hiroyuki Sumikura

**Affiliations:** 1 Department of Obstetric Anesthesia, National Center for Child Health and Development, Tokyo, Japan

I read this report with interest, as I have just experienced a case of cesarean section with a brain tumor. I wondered if spinal anesthesia was contraindicated for these patients, hence, if the author could add some discussion about this topic it would be greatly appreciated.

I have read this submission. I believe that I have an appropriate level of expertise to confirm that it is of an acceptable scientific standard.

# Response

**Published:** 2013-12-11

**Authors:** Alaa Abd-Elsayed

**Affiliations:** Community Medicine, Faculty of Medicine, Assiut University, Egypt

This is a very good point mentioned by the reviewer, we just think it is out of the scope of this article, and is a topic with a lot of controversy that really needs a full another manuscript to discuss. We focused on the good drugs and agents to use and not the technique of management.

# Peer review: Referee response for version 1

**Published:** 2013-08-27

**Authors:** Michael James Paech

**Affiliations:** 1 School of Medicine and Pharmacology, University of Western Australia, Perth, Australia

There is not much information about modern management of cerebral tumours during pregnancy, so this paper is of some interest despite the small number of cases.

**Abstract**

This draws a conclusion and makes general recommendations– yet there was no related discussion within the Discussion section of the body of the paper. It is inappropriate anyway to draw conclusions based on a small retrospective case series, so this needs revision. After describing the cases and the drugs used, I suggest the author indicates what follows next in the paper ie. a more detailed description of cases and discussion of the drugs used for general anaesthesia.

**Introduction**

This reads well and is informative. I prefer the use of “fetus” and there is a good linguistic argument to be made for this spelling. 

Guidelines should be evidence-based, or in the absence of reasonable evidence at least consensus based, from a group of experts. I don’t think it is reasonable to suggest guidelines should come from ‘studies’ or be based on single institution experience with 7 cases – perhaps the authors should suggest a plan of obstetric and neurosurgical management. 

**Results**

-  “Four patients (57%) underwent …” Please replace “case” with “patient’.
-  Remifentanil and alfentanil are misspelt in several places.
-  Table 2 legend - change to “Intracranial pressure”. Correct Table 1 heading to “Postoperative events”. 
- Delete “Only” from the last sentence.   
- Neonatal condition would be better placed in Table 2, because Table 1 describes several patients in whom tumour resection was much later than delivery, and Table 2 has the obstetric data. 
-  The GA technique for tumour resection is mentioned but not the GA technique for CS, which becomes a source of confusion when you later discuss drugs and their effects on the developing brain etc. Can this be added? 

**Discussion**

-  A comment should be made about the exclusive use of GA. Regional block may be appropriate (and preferred by the patient) for CS if there is no evidence of raised intracranial pressure or recent seizures – in this series all patients had increased ICP or seizures. 
- A comment about the location of fetal monitoring might also be useful. 
-  Correct to “cerebral oedema” and “epidural analgesia” in Management Issues. 
-  Preoperative seizure control and the implications of anticonvulsant therapy warrant a mention.
- ‘Motherhood’ statements such as “general anaesthesia is safe to be used with intracranial tumours” should be removed. 

The discussion of propofol should be re-focused and expanded a little – this is now widely considered suitable for induction in pregnancy, and the main issues are the dose-response curve compared with thiopentone and the neonatal outcome, rather than uterine relaxation. The potential neurotoxicity is not mentioned, yet later the volatiles are implicated and other drugs considered. This area of science is very interesting but no clinical conclusions have been drawn as yet, and I think it has been over-emphasised throughout the author's Discussion, to the detriment of clinical studies and information about the implications of drugs during neurosurgery. 

In relation to animal neurotoxicity studies, I suggest only drawing comparisons between various drugs and balance these with clinical information. The evidence condemning isoflurane is not supported by the references, which are paediatric case reports rather than fetal exposure. Few would consider isoflurane unacceptable, despite not being ideal; nor would desflurane or sevoflurane be avoided. 

References after 17 are muddled and do not apply to the text. This needs full review. In addition there are some odd choices of reference – e.g. the case report by McCarroll on remifentanil and peripartum cardiomyopathy is used to justify a comment about anticonvulsants, and ergometrine is discussed using a reference about craniotomy (or – all perhaps the McCarroll case? – but this would still be inappropriate).

Again, suggesting that remifentanil is safe for the developing brain is premature if based only on mechanistic action considerations. Likewise use of dexmedetomidine, quoting a single case report! Please discuss mechanisms but don’t generalise. It would make more sense to discuss other drugs, some of which were surely used in the patients, in a more logical sequence (no information provided about GA technique for CS, but presumably oxytocin must have been used, possibly also nitrous oxide?). A restructure of the Discussion section with different headings should be considered.

Please do not draw conclusions about safety based on a 7 patient case series.

I have read this submission. I believe that I have an appropriate level of expertise to confirm that it is of an acceptable scientific standard, however I have significant reservations, as outlined above.

# Response

**Published:** 2013-12-11

**Authors:** Alaa Abd-Elsayed

**Affiliations:** Community Medicine, Faculty of Medicine, Assiut University, Egypt

- *"* *After describing the cases and the drugs used, I suggest the author indicates what follows next in the paper"*
  We have changed the conclusion as advised by the referee.
   
- *"* *I prefer the use of “fetus” and there is a good linguistic argument to be made for this spelling"*
  The spelling of "fetus" was suggested by the journal.
   
- " *I don’t think it is reasonable to suggest guidelines should come from ‘studies’ or be based on single institution experience with 7 cases*"
  We have discussed the evidence from the literature. Having 7 cases from one institute with pregnant patients with brain tumors is a reasonable number, and we are not aware of any other institutions who have published in this topic. It is a rare condition and not that common.
   
- " *Neonatal condition would be better placed in Table 2*"
  Table 2 is already busy, but we will be happy to do this if okay with the journal format.
   
- " *The GA technique for tumour resection is mentioned but not the GA technique for CS*"
  We think this is not relevant to the topic we wanted to discuss. We have corrected the manuscript by changing the number of patients to the pregnant patients who had the tumor resection during pregnancy, to make sure it is relevant to the discussion of the effects of variable anesthetics on the developing fetus.
   
- " *A comment should be made about the exclusive use of GA*"
  We just described what was done, multiple anesthesiologists managed our patients and each did it according to his beliefs and practice. We did not state we did the best management, as there are not clear guidelines for the management. And this is the message - we need to have some guidelines for managing these patients.
   
- " *A comment about the location of fetal monitoring might also be useful*"
  It is always done using external monitoring.
   
- " *Correct to “cerebral oedema” and “epidural analgesia” in Management Issues*"
  We are not sure to what correction the referee refers.
   
- " *Preoperative seizure control and the implications of anticonvulsant therapy warrant a mention*"
  These are already mentioned in the discussion section.
   
- " *The discussion of propofol should be re-focused and expanded a little – this is now widely considered suitable for induction in pregnancy, and the main issues are the dose-response curve compared with thiopentone and the neonatal outcome, rather than uterine relaxation*"
  We have modified the conclusion. Thiopentone is not widely used in our insitute.
   
- " *In relation to animal neurotoxicity studies, I suggest only drawing comparisons between various drugs and balance these with clinical information*"
  This has been mentioned in our discussion.
   
- " *...suggesting that remifentanil is safe for the developing brain is premature if based only on mechanistic action considerations. Likewise use of dexmedetomidine, quoting a single case report! Please discuss mechanisms but don’t generalise.*"
  There has not been much published on these topics, and we quoted the case reports as this is what has been published.
   
- " *Please do not draw conclusions about safety based on a 7 patient case series*"
  We have changed the conclusion.
   
- We have addressed and corrected the wordings advised by the referee. The issue with the references has also been checked.
